# *Chlamydophila pneumoniae*-associated community-acquired pneumonia in paediatric patients of a tertiary care hospital in Mexico: molecular diagnostic and clinical insights

**DOI:** 10.1038/s41598-023-48701-5

**Published:** 2023-12-06

**Authors:** Jocelin Merida Vieyra, Agustín De Colsa Ranero, Deborah Palacios Reyes, Chiharu Murata, Alejandra Aquino Andrade

**Affiliations:** 1 Laboratory of Molecular Microbiology, Instituto Nacional de Pediatria, Insurgentes Sur 3700C, Insurgentes Cuicuilco, Coyoacan, 04530 Mexico City, Mexico; 2 Department of Paediatric Infectious Diseases, Instituto Nacional de Pediatria, Mexico City, Mexico; 3 Department of Research Methodology, Instituto Nacional de Pediatria, Mexico City, Mexico

**Keywords:** Microbiology, Bacteria, Clinical microbiology, Paediatric research

## Abstract

*Chlamydophila pneumoniae* is a cause of community-acquired pneumonia (CAP) and responsible for 1–2% of cases in paediatric patients. In Mexico, information on this microorganism is limited. The aim of this study was to detect *C. pneumoniae* using two genomic targets in a real-time PCR and IgM/IgG serology assays in paediatric patients with CAP at a tertiary care hospital in Mexico City and to describe their clinical characteristics, radiological features, and outcomes. A total of 154 hospitalized patients with diagnosis of CAP were included. Detection of *C. pneumoniae* was performed by real-time PCR of the *pst* and *arg* genes. Complete blood cell count, C-reactive protein measurement and IgM and IgG detection were performed. Clinical-epidemiological and radiological data from the patients were collected. *C. pneumoniae* was detected in 25 patients (16%), of whom 88% had underlying disease (*P* = 0.014). Forty-eight percent of the cases occurred in spring, 36% in girls, and 40% in children older than 6 years. All patients had cough, and 88% had fever. Interstitial pattern on chest-X-ray was the most frequent (68%), consolidation was observed in 32% (*P* = 0.002). IgM was positive in 7% and IgG in 28.6%. Thirty-six percent presented complications. Four percent died. A high proportion showed co-infection with *Mycoplasma pneumoniae* (64%). This is the first clinical report of *C. pneumoniae* as a cause of CAP in Mexican paediatric patients, using two genomic target strategy and serology. We found a frequency of 16.2% with predominance in children under 6 years of age. In addition; cough and fever were the most common symptoms. Early detection of this pathogen allows timely initiation of specific antimicrobial therapy to reduce development of complications. This study is one of the few to describe the presence of *C. pneumoniae* in patients with underlying diseases.

## Introduction

Community-acquired pneumonia (CAP) is an acute infection of the lower respiratory tract and one of the main causes of childhood morbidity and mortality worldwide. Each year, it causes the death of about one million children younger than 5 years old. This number represents approximately 15% of all deaths in this age group, and between 90 and 95% of these deaths occur in developing countries^1^.

*Chlamydophila pneumoniae* is an obligate intracellular bacterium that causes respiratory tract infections, such as pharyngitis (5%), bronchitis (0.3–5%), sinusitis (13%), exacerbations of chronic bronchitis, and CAP^2–5^. *C. pneumoniae* is responsible for approximately 1–2% of all cases of CAP in children, mainly affecting those over 3 years of age^6^. Other reports indicate that the frequency varies between 5 and 20% in school-age children and adolescents^7^. Epidemiological studies suggest a cyclical pattern (every 4 years) of pneumonia caused by *C. pneumoniae*. In a worldwide study, the frequency of CAP due to *C. pneumoniae*, was 8% in North America, 7% in Europe, 6% in Latin America, and 5% in the Asia-Africa region^2^.

As diagnosis of *C. pneumoniae* infection is complicated, so its frequency may be underestimated. Despite its low sensitivity (50–70%), culture is still considered the gold standard. Unfortunately, *C. pneumoniae* grows slowly, and use of McCoy or HeLa cell cultures, specialized laboratory and trained personnel are necessary^2,8^. Microimmunofluorescence is the serological test of choice, but it is a complex technique, its interpretation is subjective and cross-reaction with other species of *Chlamydia* spp. also occurs. In addition, it is necessary to collect serum in the initial and convalescent phases to detect an acute infection, with sensitivity of 60–82% and specificity of 40–77%^9^.

Due to their high analytical sensitivity (50–100 fg) and specificity (100%), molecular techniques, such as real-time PCR are recommended by the Centers for Disease Control and Prevention (CDC) for diagnosis of *C. pneumoniae*^10,11^.

In addition, two or more tests are usually used to diagnose this infection as real-time PCR, specific anti-*C. pneumoniae* IgM, IgG and IgA antibodies or immunohistochemistry^12–14^. Several targets have been used to detect this microorganism including *omp*A gene,^12,15,16^, tyrosine tRNA gene in a Pan-*Chlamydia* real-time PCR^17^, 16S ribosomal RNA genes^15^ and 23S ribosomal RNA genes in a novel digital microfluidic RT-qPCR Platform^18^. The *arg* gene encodes the 147 amino acid arginine repressor protein that regulates the *gln*PQ operon which expresses a putative arginine transport system in an arginine-responsive manner. The *arg* gene has been used to detect and differentiate *C. pneumoniae* from other species of the genus *Chlamydophila*^10,17^. On the other hand, *pst* gene encodes DNA-directed RNA polymerase subunit beta, a 75 kDa protein^10,11^.

Some studies have considered that a positive PCR test is sufficient to diagnose a *C. pneumoniae* infection^13^.

Timely detection allows for administration of adequate antimicrobial treatment, as chronic or recurrent infections by *C. pneumoniae* have been associated with development of complications such as chronic obstructive pulmonary disease, asthma, arthritis, heart disease, and neurological disorders (multiple sclerosis and Alzheimer's disease)^19–21^. *C. pneumoniae* can also increase risk of lung cancer development (odds ratio 1.48–1.6)^22^.

In Mexico, information on *C. pneumoniae* as a cause of CAP in the paediatric population is limited. There is only one retrospective report that indicates involvement of *C. pneumoniae* in adolescents with asthma, in which a seroprevalence of 77.5% was found^23^.

The aim of this study was to detect *C. pneumoniae* using two genomic targets in a real-time PCR and IgM/IgG serology assays in paediatric patients with CAP at a tertiary hospital in Mexico City and additionally the study describes their clinical characteristics, radiological features and outcomes.

## Material and methods

### Location and study population

This was a prospective, single-centre study that was conducted at the Instituto Nacional de Pediatria (INP), a tertiary-care hospital in Mexico City. During the study period, November 2015 to March 2017, 154 patients < 18 years old with a diagnosis of CAP that required admission were included.

### Definitions and clinical information

Diagnosis of CAP was considered in patients who presented fever (≥ 37.5 °C), cough, polypnea, or respiratory distress and who had hypoventilation, crackles, and/or effusion by clinical examination or chest X-ray. Respiratory distress was defined per guidelines of the Infectious Diseases Society of America^24^. For the patients included in the study, the above signs and symptoms started in an outpatient setting, and the patient was not hospitalized for at least 2 weeks before the onset of symptoms. Underlying disease was defined as a medical condition that involved any organ or system and that required specialized care^25^. Acute disease was defined as a short and relatively severe course (< 1 week); chronic disease was any that continued for more than a week. Wheezing was defined as a continuous, adventitious, high-pitched sound that originated in airways narrowed by spasm, thickened mucosa, and/or luminal obstruction. Crackles were discontinuous cracking sounds caused by the passage of air through secretions of the airway due to sudden equalization of gas pressure. A decrease in the level of oxygen in the blood (SpO_2_) < 92% was recorded as desaturation.

A normal radiological pattern was defined as a chest X-ray with no abnormal findings in the lung parenchyma and pleural spaces. The interstitial pattern was defined as an image resulting from oedema and inflammation, with cellular infiltrates located predominantly in the interstitial tissue of the lung. The bronchopneumonic pattern was characterized by suppurative inflammation distributed in patches around bronchi, which may or may not be localized to a single lobe of the lung. The consolidation pattern was a homogeneous increase in the density of the parenchyma, which erased the contours of vessels and the walls of the airway and could include air bronchogram. The atelectasis represented an increased density with ipsilateral traction of the trachea and mediastinum structures. The multifocal pattern was the presence of a homogeneous increase in density at multiple sites. The overdistention pattern was defined as air trapping, with increased intercostal spaces and/or flattening of the hemidiaphragms. Pleural effusion was defined as the presence of fluid in the pleural cavity resulting from excessive transudation or exudation from the pleural surface^26,27^. The radiological patterns were independently interpreted by two different infectious diseases paediatricians.

We categorized the age range as follows: term neonatal (birth–27 days), infant (28 days–12 months), toddler (13 months–2 years), early childhood (2–5 years), middle childhood (6–11 years), and early adolescence (12– < 18 years)^28^.

### Biological samples and DNA extraction

A nasopharyngeal swab was taken from each patient with a nylon swab (FLOQ Swabs; COPAN Murrieta, CA, USA). The swab was introduced in 1.5 mL of 0.85% NaCl and transported under refrigeration (4–8 °C), as soon as possible. Samples were centrifuged at 6000*g* for 10 min. The supernatant was decanted and 180uL of ATL buffer and 20uL of proteinase K (0.5 mg/mL) were added to the pellet. Samples were incubated at 56 °C for 1.5 h. DNA was extracted with QIAamp DNA Mini Kit (Qiagen, Hilden, Germany) according to the manufacturer's instructions. The DNA was eluted in 200 μL of nuclease-free water and stored at − 20 °C until use.

Two peripheral blood samples were taken, one with EDTA for a complete blood cell count and another without anticoagulant for quantification of C-reactive protein (elevated: > 0.8 mg/L) and detection of IgM and IgG by ELISA. Haematological results were interpreted based on the age range of the patient^29^.

### *C. pneumoniae* DNA standard curves

By using the reported sequence of *C. pneumoniae* ATCC 53592D, primers were designed with the program Primer3 (4.0.0)^30–32^ for PCR amplification of a fragment of the *pst* gene (F: ACATCCATAACGACGCCTTC; R: GGGTGATGTGATTGCTGATG; 456 bp) and the *arg* gene (F: CGGCAACTCAGGAGGAATTA; R: TTTGCAATCGAAACCATCAA; 361 bp). The reaction was performed using an AB9700 thermocycler (Applied Biosystems, Foster City, CA, USA) with 12.5 mL AmpliTaq Gold^®^ 360 MasterMix (Applied Biosystems, Foster City CA, USA) and 0.4 pmol/μL primers. Fragments were purified with the QIAquick PCR purification kit (Qiagen, Hilden, Germany) and cloned into the pDrive vector (Qiagen, Hilden, Germany) according to the manufacturer's instructions. To determine the real-time PCR detection limit, dilutions of recombinant plasmids of the *pst* and *arg* genes (10^7^–10^0^ DNA molecules/µL) were prepared; reactions were performed using an ABI-7500-FAST (Applied Biosystems, Foster City, CA, USA). The reaction mixture included 12.5 μL TaqMan Universal Master Mix II with UNG (Applied Biosystems, Foster City, CA, USA), 0.1 μM each primer for *pst* and *arg*, TaqMan probes for *pst* (0.2 μM) and *arg* (0.1 μM), and 4 mL DNA. We utilized primers and probes previously reported^10,11^.

### Detection of *C. pneumoniae* in respiratory samples

Detection of *C. pneumoniae* was performed for the *pst* and *arg* genes using real-time PCR with previously reported primers and probes^10,11^. The human RNase P gene was used as a DNA extraction and amplification control^33^. The reaction mixture consisted of 12.5 μL TaqMan Universal Master Mix II with UNG (Applied Biosystems, Foster City, CA, USA), 0.1 μM each primer for *pst* and *arg*, 0.3 mM primers for RNase P, TaqMan probes for *pst* (0.2 µM), *arg* and RNase P (0.1 µM), and 4 µL DNA. The reactions were performed in triplicate using an ABI-7500-FAST (Applied Biosystems, Foster City, CA, USA).

### Serological test

Anti-*C. pneumoniae* IgM and IgG antibodies were detected in 10 µL of serum with anti-*C. pneumoniae* IgM-IgG human ELISA kits (Abcam, Cambridge, UK) according to the manufacturer's instructions. The results were interpreted as follows: positive: > 11 standard units; indeterminate: 9–11; and negative: < 9.

### Statistical analysis

The demographic characteristics, such as gender and age of the patients were described using both absolute frequencies and percentages, as well as presented in terms of medians and ranges. Furthermore, the distribution of underlying diseases, clinical symptoms, and radiographic findings was compared between *C. pneumoniae*-positive and *C. pneumoniae*-negative patients employing χ^2^ or Mann–Whitney U tests. Effect sizes were determined using the Phi index or Kramer's v index in the case of χ^2^ test comparisons and the r index for comparisons conducted through the Mann–Whitney U test. Effect sizes were categorized as small (S), medium (M), or large (L) based on the criteria "0.1," "0.3," and "0.5," respectively, with values below 0.1 considered presenting null effect (N). A significance level of p < 0.05 was employed for statistical tests. Data analysis was executed utilizing the R software and the functions provided by the R package "effectize"^34–36^.

### Ethical approval

This study was approved by the Research and Ethics Committees of the INP (IRB: 00008064, IRB: 00008065) with registration number 2014/058; following the guidelines of the Declaration of Helsinki. Written informed consent was obtained from the parent or guardian of each child enrolled in the study. Patient data were deidentified.

## Results

The detection limit of the real-time PCR was 100 DNA molecules/μL for the *pst* [cycle threshold (Ct) 34.407] and *arg* (Ct 34.361) genes. During the study period, 154 paediatric patients with CAP were included, and *C. pneumoniae* was detected in 25 (16.2%). The two genomic targets (*pst* and *arg*) were amplified in 13 samples (8.5%), the *pst* gene alone in three (1.9%), and the *arg* gene alone in nine (5.8%). RNase P gene amplification was obtained in all samples (median Ct. 28.1, range 19.1–35) (Table 1).

**Table 1:** Underlying disease and detection of *C. pneumoniae* in paediatric patients with CAP.

Key	Sex	Age group	Underlying disease	*C. pneumoniae* qPCR (Ct)	
				*pst*	*arg*
AT-1	F	Older infant	None	ND	**34.287**
AT-2	F	Adolescent	GERD, chronic lung disease	ND	**28.741**
AT-3	M	Preschool	None	ND	**31.187**
AT-4	M	Preschool	Down syndrome	ND	**27.111**
AT-6	M	Older infant	Macrocephaly, congenital heart disease, neurodevelopmental delay	ND	**30.839**
AT-8	F	Younger infant	Gastroschisis	ND	**34.129**
AT-10	F	Adolescent	Acute lymphoblastic leukaemia	36.394	**33.226**
AT-14	M	School	Acute lymphoblastic leukaemia	ND	**30.811**
AT-15	M	School	Right hemiparesis, vasculitis	ND	**32.08**
AT-33	M	School	Gastroschisis, cryptorchidism	**24.317**	36.136
AT-34	M	Adolescent	Hypogammaglobulinemia	**24.29**	35.222
AT-35	M	Preschool	Chronic kidney failure	**29.013**	**25.684**
AT-36	M	School	None	**33.567**	**30.632**
AT-38	F	Younger infant	Myeloradiculitis	**34.38**	**31.429**
AT-39	F	Older infant	Congenital heart disease, hypogammaglobulinaemia	**32.124**	**28.306**
AT-41	F	Preschool	Cyclical neutropaenia	**33.769**	**29.636**
AT-44	M	Older infant	Global neurodevelopmental delay	**30.297**	**28.806**
AT-46	M	School	AIDS/HIV infection	**33.177**	**32.282**
AT-47	M	Older infant	Acute lymphoblastic leukaemia, Fanconi anaemia	**33.627**	**30.304**
AT-53	M	Younger infant	Encephalopathy, GERD, velopalatal dysfunction	**29.263**	**30.022**
AT-54	M	Older infant	Esophageal atresia	**33.743**	**33.067**
AT-55	M	Preschool	Autoimmune haemolytic anaemia	**33.286**	**31.883**
AT-60	M	School	Aortic stenosis, epilepsy	**33.64**	**31.373**
AT-61	F	Older infant	Chronic lung disease	**32.218**	**32.837**
AT-166	F	Adolescent	Follicular adenoma of the thyroid	**29.904**	34.491

*GERD* gastroesophageal reflux disease, *CAP* community-acquired pneumonia, *ND* not determined.

Positive genes by qPCR are shown in bold.

Thirty-six percent of the patients with *C. pneumoniae* were female and 64% were male. Forty percent were older than 6 years. The median age of patients with *C. pneumoniae* was 4.7 years (range 0.4–14.8 years). By age group, toddlers were the most frequent (28%), followed by middle childhood (24%) and early childhood (20%). The proportions with and without *C. pneumoniae* were similar within each age group. CAP most often occurred during spring (n = 12, 48%), followed by autumn (n = 7, 28%) (Table 2).

**Table 2:** Demographic characteristics of the patients with CAP.

Characteristic	Total, n = 154 (%)	*C. pneumoniae*		*P*	ES
		$+$ (n = 25)	$-$ (n = 129)		
Sex (female), n (%)	68 (44%)	9 (36%)	59 (46%)	0.37	0.07^N^
Age (year), median (range)	3.1 (0.04–18.3)	4.7 (0.4–14.8)	3.0 (0.04–18.3)	0.13	0.12^S^
Older than 6 years, n (%)	42 (27%)	10 (40%)	32 (25%)	0.12	0.13^S^
Age group, n (%)				0.37	0.19^S^
Term neonatal (birth-27 days)	1 (0.6%)	0 (0%)	1 (0.8%)		
Infant (28 days-12 months)	35 (23%)	3 (12%)	32 (25%)		
Toddler (13 months-2 years)	32 (21%)	7 (28%)	25 (19%)		
Early childhood (2–5 years)	44 (29%)	5 (20%)	39 (30%)		
Middle childhood (6–11 years)	22 (14%)	6 (24%)	16 (12%)		
Early adolescence (12– < 18 years)	20 (13%)	4 (16%)	16 (12%)		

*CAP* community-acquired pneumonia, *ES* effect size, *N* none, *S* small.

*P < 0.05, statistically significant.

A total of 88% of the patients with *C. pneumoniae* had underlying diseases (*P* = 0.014), mainly congenital (36%) and neurological (16%) (Table 1).

The course of the disease was acute in 56% of the patients. All patients had cough, 88% had fever, and 48% had rhinorrhoea. The median duration of cough was 5 days (range 1–90 days), and for fever was 2.5 days (range 1–10 days). Eight percent presented with headache, and 24% experienced vomiting. Regarding respiratory signs, 93% presented with desaturation, 52% with wheezing, and 68% with crackles. None of these symptoms or clinical signs showed statistically significant associations with *C. pneumoniae* (Table 3). Thirty per cent of the patients received community treatment with antibiotics, mainly beta-lactams (Table 3). The median duration of illness was 22 days (range 1–98). Median hospital stay was 16 days (range 2–42). For the radiological findings, the interstitial pattern was the most frequent (68%); however, consolidation was significantly associated with infection (32%; *P* = 0.002). Twenty-eight percent showed a bronchopneumonic pattern, and 16% had overdistention. Twelve patients presented a single radiological pattern, and 13 with mixed pattern (Table 3).

**Table 3:** Clinical data, radiological presentation and community treatment of the patients with CAP due to *C. pneumoniae.*

Key	Signs	Symptoms	X-ray chest	Complications	Community treatment	Schemes	Community treatment	Duration illness	LHS
AT-1	Crackles, wheezing	Fever, rhinorrhoea, diarrhoea	Interstitial, consolidation	Mechanical ventilation, PICU	Y	1	DX (3)	15	14
AT-2	Crackles	Fever, headache	Interstitial, consolidation	None	Y	1	CXM (1)	29	29
AT-3	Hypoventilation	Fever, pharyngitis, vomiting	Interstitial	Pericarditis	Y	4	CLR (8), CEC (7), SXT (4), AM (7)	35	10
AT-4	Wheezing, crackles	Fever	BMN	None	Y	2	CRO (4), CFM (10)	22	9
AT-6	Wheezing, hypoventilation	Fever	Interstitial, consolidation, overdistention	None	N			11	8
AT-8	Wheezing, crackles	Fever, rhinorrhoea	Consolidation, atelectasis, overdistention	None	N			28	26
AT-10	Tachycardia	Fever	Interstitial	None	N			22	21
AT-14	Tachycardia	Fever	Interstitial, overdistention	None	N			23	23
AT-15	Tachycardia, wheezing, crackles	Abdominal pain, vomiting	Interstitial, overdistention	None	Y	2	AMX (3), CRO (1)	36	16
AT-33	Tachycardia, crackles	Fever, rhinorrhoea	Interstitial, consolidation,	Mechanical ventilation, PICU	N			45	42
AT-34	Crackles	Fever, headache, vomiting	Interstitial, BMN	None	Y	1	AMC (7)	17	12
AT-35	Crackles	Fever, rhinorrhoea	Interstitial	None	N			11	11
AT-36	None	Fever, rhinorrhoea, pharyngitis, abdominal pain, vomiting	Consolidation	Mechanical ventilation, PICU, Dead	Y	1	Unknown	17	4
AT-38	Wheezing, hypoventilation	Fever, rhinorrhoea, vomiting	Interstitial, atelectasis	Mechanical ventilation, PICU	N			9	27
AT-39	Wheezing, crackles	Fever, rhinorrhoea. vomiting	BMN	Mechanical ventilation, PICU	N			39	33
AT-41	Tachycardia, wheezing, crackles	Fever, rhinorrhoea	Interstitial	None	N			18	12
AT-44	Wheezing, crackles	Rhinorrhoea	Interstitial, BMN, effusion	Mechanical ventilation, PICU	N			98	37
AT-46	Tachycardia, crackles	Fever	BMN, multiple foci	None	N			8	9
AT-47	Tachycardia, crackles	Fever, rhinorrhoea, pharyngitis,	Interstitial	None	N			6	7
AT-53	Cyanosis, crackles	Fever	BMN	None	N			18	19
AT-54	Tachycardia, wheezing, cyanosis, crackles, hypoventilation	Fever, rhinorrhoea	BMN	Mechanical ventilation, PICU	N			38	17
AT-55	Tachycardia, wheezing, crackles	Fever, rhinorrhoea, pharyngitis,	Consolidation	Mechanical ventilation, PICU, Pleural effusion	Y	1	CN (1)	32	23
AT-60	Wheezing	None	Interstitial	None	N			8	8
AT-61	Wheezing, cyanosis, crackles	Fever	Interstitial	None	N			29	28
AT-166	Hypoventilation	Fever, arthralgias	Interstitial	None	N			1	2

*CAP* community-acquired pneumonia, *BMN* bronchopneumonia, *PICU* paediatric intensive care unit, *Y* yes, *N* no, *DX* dicloxacillin, *CXM* cefuroxime, *CLR* clarithromycin, *CEC* cefaclor, *SXT* trimethoprim-sulfamethoxazole, *AM* ampicillin, *CFM* cefixime, *AMX* amoxicillin, *AMC* amoxicillin-clavulanate, *CN* cephalexin, *LHS* length hospital stay.

Complete blood count results were obtained for 23 patients with *C. pneumoniae*. A total of 17.4% had leukocytosis, 39.1% neutrophilia, 69.6% lymphopenia, and 52.2% monocytosis. C-reactive protein was measured in 20 patients: and was reported elevated (> 0.8 mg/L) in 80% (n = 16). No statistical significance was observed for any of these data.

Of the 25 patients with *C. pneumoniae*, serology was performed in 14. One patient had positive IgM and IgG, and three were only positive for IgG. In 10 patients (71.4%), no anti-*C. pneumoniae* antibodies were detected.

Thirty-six percent of the patients presented complications, most of them (n = 8) required admission to the paediatric intensive care unit (PICU) for a median of 7 days (range 3–21 days) with mechanical ventilation (median 5 days, range 3–19 days) (*P* = 0.031). In this series, two patients developed pleural effusion, one of them also presented with autoimmune hemolytic anemia. Another patient developed pericarditis and one patient died with myocarditis (4%). Fifteen patients (64%) had coinfection with *M. pneumoniae* and three with other bacteria (*Streptococcus pyogenes* n = 1, *Pseudomonas aeruginosa* and *Escherichia coli* n = 1, and methicillin-sensitive *Staphylococcus aureus* n = 1). No respiratory viral co-infections were analysed.

## Discussion

The present work describes the contribution of *C. pneumoniae* as a causal agent of CAP in Mexican paediatric patients treated in a tertiary care hospital, with a frequency of 16.2%. A retrospective study carried out in Chinese paediatric patients found a similar result (21.8%)^37^. Frequencies ranging from 0.2 to 18% have been reported for other countries^13,20,38–45^. These differences may be due to the number of patients included in each study, the diagnostic method used, and the target genes detected. The tests applied in these studies included nested PCR, serology, real-time PCR, and a combination of methods^20,38–45^. In a study from Brazil, 18% of paediatric patients with CAP were infected by *C. pneumoniae* based on real-time PCR with the 16S rRNA gene as the genomic target^20^; in Poland, 5.5% had positive serology (IgM)^38^ and 8.8% by nested PCR in Peru^40^. On the other hand, in a study from India in patients with lower respiratory tract infection (LRTI), greater positivity was found by serology (13.3%) than nested PCR (2.6%)^46^. In the present study, the *arg* gene was positive in 22 samples and the *pst* gene in 16. The real-time PCR assay using the *arg* gene has been validated by the CDC and is the recommended method for detection of *C. pneumoniae*^10^. A combination of methods might increase the probability of detection.

Of the patients with CAP due to *C. pneumoniae*, 64% were boys (*P* = 0.27). In two studies from India and Japan that included 11 and 42 patients, respectively, with LRTI due to *C. pneumoniae*, a higher frequency in males was also reported (73 and 58.8%, respectively)^46,47^. In contrast, it was more frequent in females in Korean paediatric patients (57%)^13^, whereas no sex predominance was observed in a Peruvian study^40^. It seems that the gender factor is not decisive for the infection.

Traditionally, it has been established that infections due to atypical agents occur more frequently in children over 5 years of age; however, in our study, 60% of children with *C. pneumoniae* were under 6 years of age (*P* = 0.12). Overall, data regarding age distribution differ even in the same country. For example, in China, three independent reports showed different frequencies. In a multicentre study carried out from 2008 to 2018, a frequency of 52.9% was informed in the group aged 6–10 years^47^, but a study carried out in a single-centre hospital from January 2019 to December 2020 reported that *C. pneumoniae* was predominant in children older than 6 years (64.8%)^48^. In a third study that included 81 patients with *C. pneumoniae*, patients were most often under 1 year of age (49.4%)^49^. In Peru, *C. pneumoniae* predominated in the group of 29 days to 2 months old (26.8%), followed by the group of 1 to 5 years (25.4%)^40^. In India, analysis of 11 patients with LRTIs due to *C. pneumoniae* revealed predominance in children aged two to six months (90.9%)^46^. These data indicate that unlike other infectious agents that cause CAP or LRTI, such as viruses, which mainly affect children aged 4 months to 5 years^50^, *Streptococcus pneumoniae*, which mainly affects children under 5 years^51^, and *M. pneumoniae*, that mainly affects children under 6 years^52^, *C. pneumoniae* is equally likely to impact any age group.

The seasonality of *C. pneumoniae* infection has not been established. In this study, it was most often found in spring (48%). In a Chinese study, a higher frequency was reported during winter (34.3%)^37^; in other cohorts, a uniform distribution of *C. pneumoniae* throughout the year was reported^38,40,48^.

There is limited information on *C. pneumoniae* as a cause of CAP in paediatric patients with underlying diseases^39^. In our study, 88% had a previous pathology, which was significantly higher than expected (*P* = 0.014). Congenital and neurological diseases were the most frequent, but they were not significantly more common. This situation occurs from the nature of our hospital: because it is a tertiary-care hospital, patients are referred from institutions with a lower level of specialization, which allowed us to see an epidemiological and clinical panorama of patients with comorbidities and disease development that differed from that in those healthy children with CAP. To our knowledge, only one study has been conducted in a paediatric population with CAP in Vietnam, in which respiratory and cardiac malformations were found to be risk factors for developing severe CAP (odds ratio = 11.1)^39^. It is necessary to carry out more studies in the paediatric population with underlying diseases to determine whether such conditions predispose acquisition of CAP by *C. pneumoniae* or represent a risk for complications.

It is important to remark that the clinical signs and symptoms of *C. pneumoniae* infection are nonspecific and do not differ significantly from those caused by other agents as respiratory viruses and atypical pathogens, such as *M. pneumoniae*. In our study, the main symptoms included cough (100%, *P* = 0.174) and fever (88%, *P* = 0.955) were the most frequent, in accordance with other studies^38,53^. In contrast, only 52% of Korean children with *C. pneumoniae* CAP developed fever^13^. In our study, 48% of the patients had rhinorrhoea, but this was not statistically significant (*P* = 0.772), contrary to what was reported in a study carried out in 71 Peruvian children with acute respiratory infections. (87.3%, *P* = 0.01)^40^. This can be explained of wider selection criteria, where upper and lower respiratory infections were included, contrary to our study where only CAP diagnoses were selected, but could start with a upper tract involvement.

The frequencies of wheezing (52%, *P* = 0.218) and crackles (68%, *P* = 0.402) were similar to those reported in Indian children with LRTI due to *C. pneumoniae* (46.1% and 53.8%, respectively)^53^. In studies from China and Peru, wheezing was found in 38.3 and 38% of patients with LRTI, respectively^40,49^. All these findings support the nonspecific clinical manifestations in *C. pneumoniae* infection, which strengthens the proposal to add molecular detection for diagnosis.

Of the radiological findings, the interstitial pattern was the most frequent among patients with *C. pneumoniae* (68%, *P* = 0.562). In a Polish study, this pattern was reported in 88% of children^38^, though it was reported in only 18.1% of Indian children with LRTI^46^. In this study, the consolidation pattern was statistically significant (32%; *P* = 0.002) and was found with greater frequency than that reported in Chinese children with acute respiratory tract infection (10.6%)^49^. Recently in Korea, 40% of the patients with pneumonia caused by *C. pneumoniae* showed segmental/lobar consolidation, and it was more common in older children (> 13 years old)^13^. In the same study, the bronchopneumonic pattern was the main finding (62%)^13^; in our study, it was found only in 28% of patients. On the other hand, 27.2% (n = 3) of Indian patients with LRTI due to *C. pneumoniae* had no abnormalities on radiography^46^. These patterns are also common in other acute infections such as interstitial for viral infections or consolidation in *S. pneumoniae*, therefore, there is no specific radiological pattern indicative of *C. pneumoniae* infection.

In general, correct interpretation of the complete blood cell count and inflammation markers can lead to suspicion of an acute viral or bacterial infection, as occurs with *S. pneumoniae* and the presence of neutrophilia or an increase in lymphocytes in viral infections. However, there are not such clear findings with *C. pneumoniae*. In our study, 17.4% of the patients had leukocytosis, and 80% had elevated CRP, but these differences were not statistically significant. In studies carried out in children from Brazil and China, no association was found between leukocyte values and CRP with infection by *C. pneumoniae*^20,49^. Normal levels of leukocytes have been described in patients with *C. pneumoniae*, but an increase in the count may be observed in severe cases^54^.

Of the 14 patients for whom serology was performed, only four had anti-*C. pneumoniae* antibodies (28.6%). In the rest, the presence of underlying disease or an early stage of the disease might have affected production of antibodies, and it has been described that IgM may appear at 2–3 weeks after the onset of symptoms^55^. As mentioned above, our hospital cares mainly for the paediatric population with underlying pathologies, which can interfere with the immune response; thus, serology may not be the optimal method for detection of *C. pneumoniae* in these patients.

Information on *C. pneumoniae* and the development of complications is scarce. In Spain, of 84 paediatric patients with CAP due to atypical bacteria, 20.2% were admitted to the PICU^43^. In our study, the most frequent complication was admission to the PICU and mechanical ventilation (32%). Of these patients, six had underlying disease (mainly congenital), which might have influenced the severity of the clinical presentation.

Regarding relevant complications, two patients developed pleural effusion. One of them, a 25-month-old and previously healthy male, presented with a significant hematological alteration, characterized by severe anemia (hemoglobin 3.6 gr/dl and thrombocytopenia (51,000) as well as leukocytosis (47,000 cells, 45,000 neutrophils), a neoplastic process was ruled out, but a Cold-reactive autoimmune hemolytic anemia (cAIHA) was confirmed, characterized by hemolysis, a positive Coombs test and positive C3b. In this case other viruses were negative. The effusion had characteristics of empyema, required pleural drainage and decortication by open thoracic surgery.

It has been described that cAIHA is usually associated with infections, like *M. pneumoniae* or Epstein–Barr virus (EBV) infection, measles, varicella, influenza and recently with COVID-19 and SARS-CoV-2 vaccination^56,57^. Because this patient had a co-infection with *M. pneumoniae*, we could not determine which of the two agents triggered the cAIHA.

A second patient with pleural effusion was a 10-month-old male who had a neurodevelopmental disorder due to perinatal hypoxia, spastic paralysis, and severe malnutrition. He had a very prolonged course of respiratory symptoms, with multiple outpatient antimicrobial treatments. He developed complicated pneumonia with empyema and a tracheobronchial fistula, requiring thoracentesis, partial pneumonectomy, and tracheostomy. This patient was also reported with *M. pneumoniae* co-infection.

We also report a 30-month-old patient who was admitted for febrile syndrome, pneumonia, and cardiomegaly, after ruling out structural heart disease, moderate pericarditis was documented by transthoracic echocardiography; this effusion did not require pericardial drainage. Viral agents were ruled out as a cause of pericarditis, but we also found that this patient had co-infection with *M. pneumoniae.*

The presence of pericarditis and even hemorrhagic pericarditis is a well-established entity caused by *M. pneumoniae* or *C. pneumoniae*. In a recent systematic review, only six cases of pericarditis associated with *C. pneumoniae* were reported, of which half of them were paediatric patients^58^. In this case, given the co-infection with both pathogens and to the fact that pericardial fluid was not obtained, it was not possible to determine which of the two pathogens or both contributed to the development of the pericardial effusion.

Mortality ranges from 3.7 to 15% in patients with CAP due to *C. pneumoniae*. In Brazil, a mortality rate of 3.7%^20^ was reported, and it was 15% in Thai children^42^. In this series, one death was reported, a previously healthy 11-year-old male adolescent, who developed a basal pneumonia, with myocarditis leading to cardiogenic shock and complete AV block, who died 72 h after his admission for the cardiac complication. This patient also had co-infection with *M. pneumoniae.* Cardiac involvement due to *C. pneumoniae* is well established, however, the reported cases of myocarditis due to this agent in paediatric age are limited; like the other cases described in this series, it is not possible to establish a direct causal relationship of *C. pneumoniae* given the co-infection with *M. pneumoniae* that causes myocarditis as well^59^ This highlights the low availability of diagnostic methods for atypical bacteria in hospitals in our country, as well as the lack of diagnostic suspicion on the part of clinical staff.

One of the most important findings that we detected in this study is the association with *M. pneumoniae*, because 15 patients had coinfection with this pathogen (60%). In Peru, this coinfection was detected in three of 146 patients (2.06%)^60^; in Chinese children with RTI, it was reported in 36.2% of 724 patients^37^. Another study in Japan reported the simultaneous presence of the two agents in 0.4% of patients, which was associated with a higher proportion of fever (100%) than infection by *C. pneumoniae* alone (50%)^47^. In a Korean study, *Haemophilus influenzae* was the main coinfecting bacterium (62%) followed by *S. pneumoniae* (29%)^13^. Recently, coinfection with *C. pneumoniae* or *M. pneumoniae* was found in 7.7% of children infected with SARS-CoV-2. This coinfection required that 26% of patients be admitted to the PICU, whereas only 2.7% of those who were only infected with SARS-CoV-2 required intensive care in the PICU^61^. These results show that simultaneous infection of *C. pneumoniae* with another pathogen can influence the clinical presentation and lead to a more severe course of the disease. Detection of these coinfections is improved by molecular techniques detecting multiple respiratory pathogens^62^. In this study, it was not possible to detect co-infection with other respiratory viruses.

Our work has some limitations. Because this study was carried out only in a reference hospital centre, it does not represent the complete epidemiology of CAP due to *C. pneumoniae* in our country. The INP is a third-level hospital that concentrates the population that is referred from other first- and second-level centres, mainly involving patients with underlying conditions. On the other hand, by clinical condition it was not possible to obtain a blood sample from all patients to perform the complete blood cell count, C-reactive protein measurement, and anti-*C. pneumoniae* antibody detection.

In the future, we consider to perform a multicentre study in Mexico that includes healthy paediatric population, on the other hand, its necessary to know the role of *C. pneumoniae* in extrapulmonary conditions and in patients with asthma.

## Conclusions

This study represents the first series report in Mexico of *C. pneumoniae* as a cause of CAP in paediatric patients. It was found in 16.2% of our CAP patients, mainly in children under 6 years of age. This study is one of the few to describe the presence of *C. pneumoniae* in patients with underlying diseases. Thirty-six percent of the patients presented complications. And we found a high co-infection with *M. pneumoniae*. Timely detection of this pathogen using molecular techniques allows early administration of appropriate antimicrobial treatment and thus reduces development of associated complications.

## Data Availability

All the data supporting our findings are contained within the manuscript.

## Abbreviations

CAP: Community-acquired pneumonia
CDC: Centers for Disease Control and Prevention
CRP: C-reactive protein
INP: Instituto Nacional de Pediatria
LRTI: Lower respiratory tract infection
PICU: Paediatric intensive care unit
cAIHA: Cold-reactive autoimmune hemolytic anemia
EBV: Epstein–Barr virus
IRB: Institutional Review Board

## References

[1] DeAntonio R (2016). Epidemiology of community-acquired pneumonia and implications for vaccination of children living in developing and newly industrialized countries: A systematic literature review. Hum. Vaccin. Immunother..

[2] Burillo A, Bouza E (2010). *Chlamydophila pneumoniae*. Infect. Dis. Clin. N. Am..

[3] Park JY (2016). Microorganisms causing community-acquired acute bronchitis: The role of bacterial infection. PLoS ONE..

[4] Robinson JL (2021). Paediatrics: how to manage pharyngitis in an era of increasing antimicrobial resistance. Drugs Context..

[5] Sawada S, Matsubara S (2021). Microbiology of acute maxillary sinusitis in children. Laryngoscope..

[6] Shim JY (2020). Current perspectives on atypical pneumonia in children. Clin. Exp. Pediatr..

[7] Kogan, R & Maggiolo, J. Atypical pneumonia. In *Pediatric Respiratory Diseases* (eds. Bertrand, P. & Sánchez, I.). 309–321 (Springer, 2020).

[8] Hammerschlag, M.R., Kohlhoff, S.A. & Gaydos, C.A. *Chlamydia pneumoniae* in *Mandell, Douglas, and Bennett's Principles and Practice of Infectious Diseases* (eds. Bennett, J.E., Dolin, R. & Blaser, M.J.). 2174–2182 (W.B. Saunders, 2015).

[9] Benitez AJ (2012). Comparison of real-time PCR and a microimmunofluorescence serological assay for detection of *Chlamydophila pneumoniae* infection in an outbreak investigation. J. Clin. Microbiol..

[10] Thurman KA, Warner AK, Cowart KC, Benitez AJ, Winchell JM (2011). Detection of *Mycoplasma pneumoniae*, *Chlamydia pneumoniae*, and *Legionella* spp. in clinical specimens using a single-tube multiplex real-time PCR assay. Diagn. Microbiol. Infect Dis..

[11] Ling CL, McHugh TD (2013). Rapid detection of atypical respiratory bacterial pathogens by real-time PCR. Methods Mol. Biol..

[12] Jama-Kmiecik A (2019). Atypical and typical bacteria in children with community acquired pneumonia. Adv. Exp. Med. Biol..

[13] Han HY (2023). Surge of *Chlamydia pneumoniae* pneumonia in children hospitalized with community-acquired pneumonia at a single center in Korea in 2016. J. Infect. Chemother..

[14] de Higuchi ML (2003). Coinfection with *Mycoplasma pneumoniae* and *Chlamydia pneumoniae* in ruptured plaques associated with acute myocardial infarction. Arq. Bras. Cardiol..

[15] Stivala A (2020). Comparison of cell culture with three conventional polymerase chain reactions for detecting *Chlamydophila pneumoniae* in adult's pharyngotonsillitis. Curr. Microbiol..

[16] Jiang XW (2022). Development of a diagnostic assay by three-tube multiplex real-time PCR for simultaneous detection of nine microorganisms causing acute respiratory infections. Sci. Rep..

[17] Wolff BJ (2023). Multiplex real-time PCR assay for the detection of all *Chlamydia* species and simultaneous differentiation of *C. **psittaci* and *C. pneumoniae* in human clinical specimens. Ann. Lab. Med..

[18] Huang H (2022). A digital microfluidic RT-qPCR platform for multiple detections of respiratory pathogens. Micromachines (Basel)..

[19] Porritt RA, Crother TR (2016). *Chlamydia pneumoniae* infection and inflammatory diseases. For. Immunopathol. Dis. Ther..

[20] Alves MS (2020). High frequency of *Chlamydia pneumoniae* and risk factors in children with acute respiratory infection. Braz. J. Microbiol..

[21] Piekut T (2022). Infectious agents and Alzheimer's disease. J. Integr. Neurosci..

[22] Premachandra NM, Jayaweera J (2022). *Chlamydia pneumoniae* infections and development of lung cancer: Systematic review. Infect. Agent Cancer.

[23] Garcia G (2021). Exposure to antibodies anti-*Chlamydophila pneumoniae* associated to respiratory symptoms of asthma among adolescents. Med. Res. Arch..

[24] Bradley JS (2011). Pediatric Infectious Diseases Society and the Infectious Diseases Society of America. The management of community-acquired pneumonia in infants and children older than 3 months of age: Clinical practice guidelines by the Pediatric Infectious Diseases Society and the Infectious Diseases Society of America. Clin. Infect. Dis..

[25] Lindley LC, Cozad MJ, Fortney CA (2020). Pediatric complex chronic conditions: Evaluating two versions of the classification system. West. J. Nurs. Res..

[26] Bertrand, P. Clinical history and physical examination of the respiratory system. In *Pediatric Respiratory Diseases* (eds. Bertrand, P. & Sánchez, I.). 29–36 (Springer, 2020).

[27] García Bruce, C., Parra Rojas, R. Study of images in respiratory diseases. In *Pediatric Respiratory Diseases* (eds. Bertrand, P. & Sánchez, I.).107–126 (Springer, 2020).

[28] Williams K (2012). Standard 6: Age groups for pediatric trials. Pediatrics..

[29] Ahsan, S. & Noether N. J. Hematología. In *Manual Harriet Lane de Pediatría: Para la Asistencia Pediátrica Ambulatoria* (eds. Robertson, J. & Johns Hopkins Hospital). 322–353 (Elsevier, 2006).

[30] Koressaar T, Remm M (2007). Enhancements and modifications of primer design program Primer3. Bioinformatics..

[31] Untergasser A (2012). Primer3—New capabilities and interfaces. Nucleic Acids Res..

[32] Koressaar T (2018). Primer3_masker: Integrating masking of template sequence with primer design software. Bioinformatics..

[33] Tatti KM, Sparks KN, Boney KO, Tondella ML (2011). Novel multitarget real-time PCR assay for rapid detection of *Bordetella* species in clinical specimens. J. Clin. Microbiol..

[34] Cohen , J. The concepts of power analysis. In *Statistical Power Analysis for the Behavioral Sciences* (ed. Cohen, J.). 1–17 (Lawrence Erlbaum Associates, 1988).

[35] The R Project for Statistical Computing. https://www.r-project.org/. Accessed 28 Oct 2021.

[36] Ben-Shachar MS, Lüdecke D, Makowski D (2020). Effect size: Estimation of effect size indices and standardized parameters. JOSS..

[37] Chen JR, Zhou XF (2020). A retrospective survey of *Chlamydia pneumoniae* infection rates in paediatric patients from a single centre in Wuxi, China. J. Int. Med. Res..

[38] Kicinski P, Wisniewska-Ligier M, Wozniakowska-Gesicka T (2011). Pneumonia caused by *Mycoplasma pneumoniae* and *Chlamydophila pneumoniae* in children—Comparative analysis of clinical picture. Adv. Med. Sci..

[39] Huong Ple T (2014). First report on prevalence and risk factors for severe atypical pneumonia in Vietnamese children aged 1–15 years. BMC Public Health..

[40] Del Valle-Mendoza J (2017). High prevalence of *Mycoplasma pneumoniae* and *Chlamydia pneumoniae* in children with acute respiratory infections from Lima, Peru. PLoS One..

[41] Gong C (2018). Distribution of the atypical pathogens of community-acquired pneumonia to disease severity. J. Thorac. Dis..

[42] Bunthi C (2019). Enhanced surveillance for severe pneumonia, Thailand 2010–2015. BMC Public Health..

[43] Otheo E (2022). Viruses and *Mycoplasma pneumoniae* are the main etiological agents of community-acquired pneumonia in hospitalized pediatric patients in Spain. Pediatr. Pulmonol..

[44] Rueda ZV (2022). Induced sputum as an adequate clinical specimen for the etiological diagnosis of community-acquired pneumonia (CAP) in children and adolescents. Int. J. Infect. Dis..

[45] Yun KW (2022). Clinical characteristics and etiology of community-acquired pneumonia in US children, 2015–2018. Pediatr. Infect. Dis. J..

[46] Kumar S, Kashyap B, Kumar S, Kapoor S (2020). Diagnostic utility of serology and polymerase chain reaction for detection of *Mycoplasma pneumoniae* and *Chlamydophila pneumoniae* in paediatric community-acquired lower respiratory tract infections. Indian J. Med. Microbiol..

[47] Oishi T (2020). Low prevalence of *Chlamydia pneumoniae* infections during the *Mycoplasma pneumoniae* epidemic season: Results of nationwide surveillance in Japan. J. Infect. Chemother..

[48] Cai F, Shou X, Ye Q (2022). Epidemiological study on *Mycoplasma pneumoniae* and *Chlamydia pneumoniae* infection of hospitalized children in a single center during the COVID-19 pandemic. Front. Cell. Infect. Microbiol..

[49] Chen Z (2013). Epidemiology and associations with climatic conditions of *Mycoplasma pneumoniae* and *Chlamydophila pneumoniae* infections among Chinese children hospitalized with acute respiratory infections. Ital. J. Pediatr..

[50] Cardinale F, Cappiello AR, Mastrototaro MF, Pignatelli M, Esposito S (2013). Community-acquired pneumonia in children. Early Hum. Dev..

[51] Thadchanamoorthy V, Dayasiri K (2021). Review on pneumococcal infection in children. Cureus.

[52] Merida-Vieyra J (2019). Detection of *Mycoplasma pneumoniae* in Mexican children with community-acquired pneumonia: Experience in a tertiary-care hospital. Infect. Drug Resist..

[53] Kumar S, Saigal SR, Sethi GR, Kumar S (2016). Application of serology and nested polymerase chain reaction for identifying *Chlamydophila pneumoniae* in community-acquired lower respiratory tract infections in children. Indian J. Pathol. Microbiol..

[54] Wang X., Li H. & Xia Z. *Chlamydia pneumoniae* pneumonia. In *Radiology of Infectious Diseases* (ed. Li, H.). 69–74 (Springer, 2015).

[55] Miyashita N (2015). Antibody responses of *Chlamydophila pneumoniae* pneumonia: Why is the diagnosis of *C. pneumoniae* pneumonia difficult?. J. Infect. Chemother..

[56] Voulgaridou A, Kalfa TA (2021). Autoimmune hemolytic anemia in the pediatric. Setting J. Clin. Med..

[57] Fattizzo B, Pasquale R, Bellani V, Barcellini W, Kulasekararaj AG (2021). Complement mediated hemolytic anemias in the COVID-19 era: Case series and review of the literature. Front. Immunol..

[58] Kyriakoulis K (2021). G *et al*, *Chlamydia pneumoniae*-associated pleuropericarditis: A case report and systematic review of the literature. BMC Pulm. Med..

[59] Li CM (2013). Age-specific *Mycoplasma pneumoniae* pneumonia-associated myocardial damage in children. J. Int. Med. Res..

[60] Del Valle-Mendoza J (2017). Molecular etiological profile of atypical bacterial pathogens, viruses and coinfections among infants and children with community-acquired pneumonia admitted to a national hospital in Lima, Peru. BMC Res. Notes.

[61] Yakovlev AS (2022). SARS-CoV-2 infection in children in Moscow in 2020: Clinical features and impact on circulation of other respiratory viruses: SARS-CoV-2 infection in children in Moscow in 2020. Int. J. Infect. Dis..

[62] Tazi S (2022). Comparative performance evaluation of FilmArray BioFire RP2.1 and MAScIR 2.0 assays for SARS-CoV-2 detection. Adv. Virol..

